# Visibility and hotspots of outdoor tobacco advertisement around educational facilities without an advertising ban: Geospatial analysis in Surabaya City, Indonesia

**DOI:** 10.18332/tpc/112462

**Published:** 2019-10-04

**Authors:** Hario Megatsari, Ilham A. Ridlo, Vilda Amir, Dian Kusuma

**Affiliations:** 1 Department of Health Promotion and Behavior Sciences, Faculty of Public Health, Universitas Airlangga, Surabaya, Indonesia; 2 Department of Health Policy and Administration, Faculty of Public Health, Universitas Airlangga, Surabaya, Indonesia; 3 Faculty of Public Health, Universitas Indonesia, Depok, Indonesia; 4 Centre for Health Economics and Policy Innovations, Imperial College Business School, London, United Kingdom

**Keywords:** visibility, hotspots, outdoor tobacco advertisements, educational facility, Indonesia

## Abstract

**INTRODUCTION:**

Despite having over 60 million smokers in 2018, Indonesia still lacks tobacco control measures, including an outdoor tobacco advertising ban. This study aimed to provide evidence on the visibility and hotspots of advertisements around educational facilities in a city without a ban.

**METHODS:**

We collected data on the locations of outdoor tobacco advertisements and schools and universities in Surabaya city. We conducted buffer and hotspots analyses using ArcMap. Using Getis-Ord Gi* statistics, hotspot analysis identifies significant clusters with a high number of advertisements.

**RESULTS:**

We found 307 large and medium-sized outdoor tobacco advertisements and 1287 educational facilities (1199 schools, 88 universities). Almost 80% of those advertisements (237 units) were just 300 m away (10-minute walk) from primary schools and high schools in the city. More than half of all schools (652) and two-thirds of all universities (59) were inside hotspots where there were statistically significant clusters with a high number of advertisements. These hotspots were more densely populated and more-deprived areas.

**CONCLUSIONS:**

There was high visibility of large and medium-sized outdoor tobacco advertisements around educational facilities in the city without the ban.

## INTRODUCTION

Indonesia is among the main contributors to global smokers, with 61.4 million current smokers in 2018^1^. The latest national health survey showed that the smoking prevalence among adults aged ≥15 years remained high at 34% in 2018 and that among youth aged 10–18 years it increased from 7.2% to 9.1% in the period 2013–2018^2^. The proportion of adult smokers, who started very early at age 5–9 years, more than doubled from 0.6% to 1.5% in the period 1995–2013, while those who began at age 10–14 years increased from 9% to 17.3% during the same period^3^.

One main factor is the lack of comprehensive national tobacco control. Indonesia is still not among the 181 countries that have signed and ratified the Framework Convention on Tobacco Control (FCTC), which provides legal support for comprehensive efforts^4^. At the national level, there are currently no bans on direct advertisements, point-of-sale advertisements, and product displays. The flagship national program has been the Presidential Decree 109/2012, which encourages local governments to implement a smoke-free policy in selected facilities including those of health, education, and workplace. In these facilities, producing, selling, advertising, promotion and smoking of tobacco products are prohibited. Recent studies, however, showed only 67% of districts (345/514) adopted the policy as of 2018, with considerable variations in compliance rates from 17% in Jayapura to 78% in Bogor city^5^ (also Wahyuti W, et al, unpublished data, 2019).

Since cigarette advertising is causally linked to cigarette use among youth^6,7^, many countries have enforced the national ban on outdoor tobacco advertising including the United States (1998), United Kingdom (2003), and Sri Lanka (2006). While there is no such ban at the national level in Indonesia, few districts (15/514) have made the effort, but there are implementation issues such as enforcement and compliance. Banyuwangi district enacted the ban on main roads and sports arenas in 2016, but a survey found high visibility of about 1300 advertisement materials a year later^8^. There is currently no such evidence in districts without the ban.

Literature is also limited in at least two ways. First, many studies are mainly older (1990s) and from high-income countries such as the United States^9-11^. Second, since many nations have adopted a national ban on outdoor tobacco advertising since the FCTC in 2005, those studies have not employed the recently developed geospatial techniques. The hotspot analysis, for instance, that uses Getis-Ord Gi* statistics to identify clusters^12^ has been increasingly used in infectious disease epidemiology research but not much in non-communicable disease, including tobacco control^13,14^.

This study aimed to provide evidence on the visibility and hotspots of outdoor tobacco advertisements around schools and universities in Surabaya city where there is no advertising ban. Surabaya is the capital city of East Java province and the second largest city of Indonesia, with over 3 million people in 2017. It was among the first district governments to implement smoke-free areas banning indoor smoking in selected facilities including schools since 2008^15^. However, it is currently lagging in more comprehensive tobacco control measures.

## METHODS

We conducted a geospatial analysis of the visibility and hotspots of outdoor tobacco advertisements around schools and universities in Surabaya. There are two primary data: advertisement and educational facility data.

First, we collected data on large and medium-sized advertisements during October–November 2018 by surveying over 250 registered roads and streets (as per the mayor’s regulation number 70 of 2010^16^) using motorcycles and cars. Variables included geocodes (latitude and longitude), types (videoboard, billboard, and banner), brand/product name, and picture. We used Google My Location App on smartphones to obtain the geocodes^17,18^.

Second, educational facility data include a comprehensive list of government and private schools and universities in Surabaya. We obtained the school data from the website of the city education office with variables: school name, level (primary, junior high, and senior high), ownership (government, private), and address. We obtained the university data from the website of the national higher education office with similar variables. Both school and university data were as of December 2018. We used Google Sheets and geocoding addons to convert the addresses into geocodes. Also, we collected published subdistrict demographic and socioeconomic data from the Statistics Bureau of the city.

The geographical analyses were conducted in ArcMap 10.6 using the World Topographic Map as a basemap. We used several geospatial tools: 1) geoprocessing/buffer tool to generate buffers of 100 m and 300 m around advertisements^19-21^; 2) spatial join tool to calculate the number of advertisements that have at least one school within a buffer; 3) spatial join and dissolve tools to produce number of schools within an advertisement buffer; 4) kernel density tool to generate heatmap of advertisements and optimized hotspot analysis tool to produce the hot spots (defined as significant clusters with a high number of advertisements using 95% significance levels); and 5) spatial join tool to produce number of schools/universities within hot spots. In the analyses, we represented each advertisement and facility as a point on the map while government universities as polygons because of typically larger areas.

## RESULTS

Table 1 shows the descriptive statistics of outdoor tobacco advertisements and educational facilities of our analysis. There were 307 large and medium-sized advertisements, which included billboards (63%), banners (31%), and videoboards (7%) (Appendix 1 shows sampled pictures). The three most prominent companies, including local companies such as PT. Djarum and PT. Gudang Garam and global companies such as PT. HM Sampoerna (Philips Morris International) owned most of the advertisements (90%). There were 1287 educational facilities, including 1199 schools and 88 universities. Over half of the schools were at the primary school level, and two-thirds were private. Over 90% of universities were private.

**Table 1:** Outdoor tobacco advertisements and educational facilities in Surabaya city, 2018

*Categories*	*n*	*%*
**Advertisement by type**		
Billboard	193	63
Banner	94	31
Videoboard	20	7
Total	307	
**Advertisement by company**		
PT. Djarum	116	38
PT. HM Sampoerna	83	27
PT. Gudang Garam	73	24
Other companies	35	11
Total	307	
**School by type (grades)**		
Primary school (6–12)	684	57
Junior high school (13–15)	346	29
Senior high school (16–18)	169	14
Total	1199	
**School by ownership**		
Government	397	33
Private	802	67
Total	1199	
**University by ownership**		
Government	7	8
Private	81	92
Total	88	

One advert was excluded because it was outside of the city boundary used for spatial analysis. Other companies include PT. Bentoel International, PT. Wismilak, PT. Karyadibya Mahardika, PT. Kolang Citra Abadi, and PT. Nojorono Tobacco. The University of Airlangga has three separate campuses (counted as three facilities) but Sepuluh November Institute of Technology, the State Polytechnic in Electronics, and the State Polytechnic in Marine Engineering were within the same campus complex so were counted as one facility.

**Figure 1:**
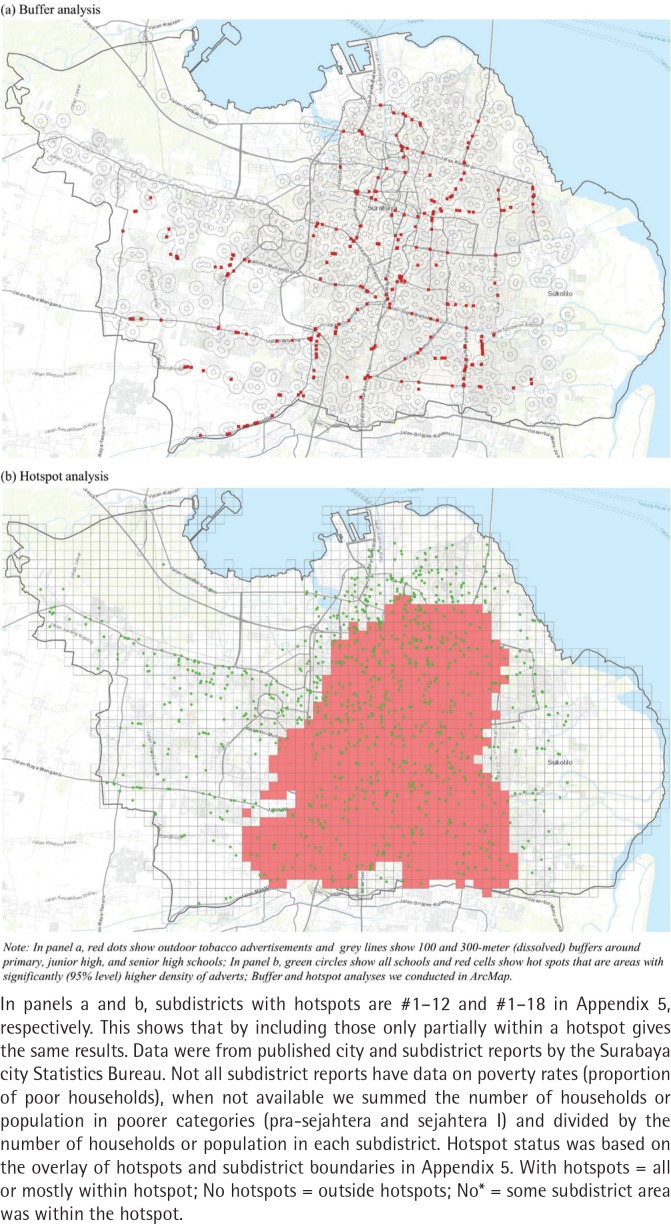
Visibility and hotspots of outdoor tobacco advert around educational facility in Surabaya, 2018

Figure 1 (panel a) shows the visibility of outdoor tobacco advertisements using buffer analysis. The red dots show outdoor tobacco advertisements and grey lines show dissolved buffers of 100 m (about 5-minute walk) and 300 m (about 10-minute walk) around schools. This result shows a high visibility of advertisements around school buffers. Table 2 shows the number of educational facilities with at least one advertisement inside school buffers and the number of advertisements within dissolved school buffers. Results show that 27% of schools (326/1199) and 31% of universities (27/88) had at least one advertisement within 300 m from the facility. By ownership, visibility was similar between government and private schools, but it was higher around government universities (57% vs 28%, of government and private universities, respectively, had at least one advertisement within 300 m). Moreover, results also show that 78% of advertisements (239/307) and 11% of advertisements (11/307) were just 300 m from schools and universities, respectively. There were more advertisements around private schools (188 advertisements or 61% of total) and around primary schools (209 advertisements or 68% of total).

**Table 2:** Number and proportion of educational facilities and outdoor tobacco advertisements related to buffers

*Educational facilities*	*Number of facilities with at least one advert within buffer*			*Number of adverts within buffer (dissolved)*			*Number of facilities within advert hotspot*	
		*100 m*	*300 m*		*100 m*	*300 m*		
	*Total*	*n (%)*	*n (%)*	*Total*	*n (%)*	*n (%)*	*Total*	*n (%)*
All schools	1199	48 (4)	326 (27)	307	54 (18)	239 (78)	1199	652 (54)
Government	397	20 (5)	113 (28)	307	27 (9)	155 (50)	397	225 (57)
Private	802	28 (3)	213 (27)	307	34 (11)	188 (61)	802	427 (53)
Primary	684	29 (4)	185 (27)	307	45 (15)	209 (68)	684	378 (55)
Junior high	346	13 (4)	92 (27)	307	22 (7)	152 (50)	346	183 (53)
Senior high	169	6 (4)	49 (29)	307	8 (3)	90 (29)	169	91 (54)
All universities	88	8 (9)	27 (31)	307	14 (5)	33 (11)	88	59 (67)
Government	7	4 (57)	4 (57)	307	10 (3)	12 (4)	7	6 (86)
Private	81	4 (5)	23 (28)	307	4 (1)	22 (7)	81	53 (65)

Advert: advertisement. Buffer analysis, hotspot analysis and calculations were conducted in ArcMap. Hotspot analysis used Getis-Ord Gi* statistics. Hotspots show a significant cluster of a higher number of tobacco advertisements at a 95% level.

Figure 1 (panel b) shows the hotspots of outdoor tobacco advertisements using hotspot analysis. The green dots are schools, and the red-shaded areas are the hotspots (i.e. areas with significantly higher density of ads). Results show that the middle part of the city has a significant (95% level) clustering of advertisements (the default ArcMap result is given in Appendix 2). This clustering is also shown in the kernel density map in Appendix 3. Table 2 shows the number and proportion of educational facilities within the hotspots. Results show high visibility around facilities, with 54% of schools (652/1199) and 67% of universities (59/88) inside the hotspot areas. Results also show that 57% and 53% of government and private schools were within hotspots, respectively, while the corresponding universities were 86% and 65%, respectively. To better understand the hotspot areas, Table 3 shows the characteristics of subdistricts with and without advertisement hotspots. We overlaid the hotspots with the subdistrict boundary map and defined subdistricts with hotspots if all or most of a subdistrict area is within the hotspot. Twelve of 31 subdistricts in the city were defined as hotspots (Appendix 4). We then summarized the demographic and socioeconomic characteristics of areas with and without hotspots (Appendix 5). Results show that the subdistricts with hotspots are, on average, more densely populated (20333 vs 7532 people per km^2^) and have higher poverty rates (21.6% vs 16.9%) compared to those without hotspots.

**Table 3:** Characteristics of subdistricts with and without advertisement hotspots

	*Subdistricts with hotspots*	*Subdistricts with no hotspots*
**Subdistrict 12**		
Population	116258	82548
Area size km^2^	6.1	13.6
Population density per km^2^	20333	7532
Poverty rates	21.6	16.9
**Subdistrict 18**		
Population	106559	80418
Area size km^2^	9.1	13.0
Population density per km^2^	15579	8207
Poverty rates	21.6	16.9

## DISCUSSION

There was high visibility of large and medium-sized outdoor tobacco advertisements around educational facilities in a city without the ban. Many of those advertisements were large billboards and videoboards, from which young people were more likely to recall information, relative to smaller advertisements^22^. Almost 80% of those advertisements were about 10 minutes away from primary schools and high schools in the city, and more than half of schools and two-thirds of universities were inside the advertisement hotspots. Also, most of the advertisements were owned by the three biggest tobacco industries in the country, which have marketed cigarettes aggressively and attractively, especially to the youth^23-25^. All this encourages receptivity and favoritism to advertisements, which has shown to increase tobacco use among young people^7,26^. Results also show significant hotspots of ads, particularly in the middle areas of the city that are shown to be more densely populated and more impoverished. This could contribute to the increasing tobacco use among poorer populations, particularly youth^27^.

For global tobacco control, all this supports and justifies the crucial role of an effective ban on outdoor tobacco advertising in reducing exposure to tobacco marketing, particularly to young people. Even in settings that have had regulations to ban outdoor tobacco advertisements, implementation issues like low enforcement and compliance are likely to arise^8^. For Indonesia, this evidence should serve as a wakeup call for the government to ban outdoor tobacco advertisements, ideally a total ban but at least a near-school ban, in order to halt the increasing trend of smoking prevalence among youth^21,28^. Currently, only 3% of districts have had some regulations to ban outdoor tobacco advertising. Concerted efforts should be made to extend the ban to the 97% (449) of the districts.

### Limitations

Our study has at least two limitations. First, it has not included smaller sized advertisements. Further research should also assess the visibility of those advertisements. Second, this study only analyzed the visibility and hotspots around schools and universities. Further studies should evaluate the visibility beyond schools, including health facilities, places of worship, and markets.

## CONCLUSIONS

There was high visibility and hotspots of large and medium-sized outdoor tobacco advertisements around educational facilities in a city without the ban in Indonesia. This evidence supports the important role of an effective prohibition of outdoor tobacco advertisements in reducing the potential exposure to tobacco marketing to young people. This evidence should also serve as a wakeup call for the national and district governments in Indonesia to ban advertisements totally or at least near schools to halt the increasing trend of smoking prevalence among youth.
