# Supplementary material for: Visibility and hotspots of outdoor tobacco advertisement around educational facilities without an advertising ban: Geospatial analysis in Surabaya City, Indonesia
Source: Tob Prev Cessat. 2019 Oct 4;5:32. doi: 10.18332/tpc/112462 (PMC7205114; doi:10.18332/tpc/112462)
Supplement: Supplementary file 1 [file TPC-5-32-s1.pdf]

SUPPLEMENTARY ONLINE APPENDIX

The authors have provided this appendix to give readers additional information about their work.

Supplement to: Visibility and hotspots of outdoor tobacco advertisement around educational facilities without an advertising ban: Geospatial analysis in Surabaya City, Indonesia

(Updated Sep 4, 2019)

Contents

Appendix 1. Sampled pictures of outdoor tobacco advertisements

Appendix 2. Hotspot analysis results using ArcMap default version

Appendix 3. Kernel density of outdoor tobacco advertisements

Appendix 4. Overlay outdoor tobacco advert hotspots and subdistrict boundaries

Appendix 5. Characteristics of subdistricts and hotspot status

Appendix 1. Sampled pictures of outdoor tobacco advertisements

(a) 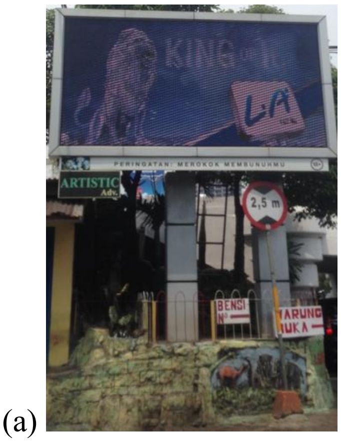

(b) 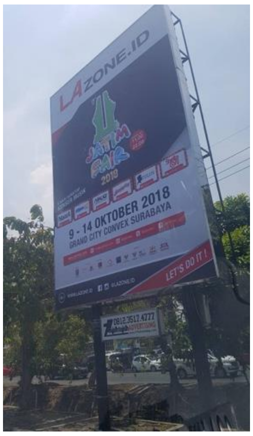

(c) 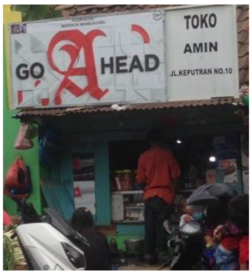

Note: (a) Videotron (electronic display), (b) billboard, and (c) banner

Appendix 2. Hotspot analysis results using ArcMap default version

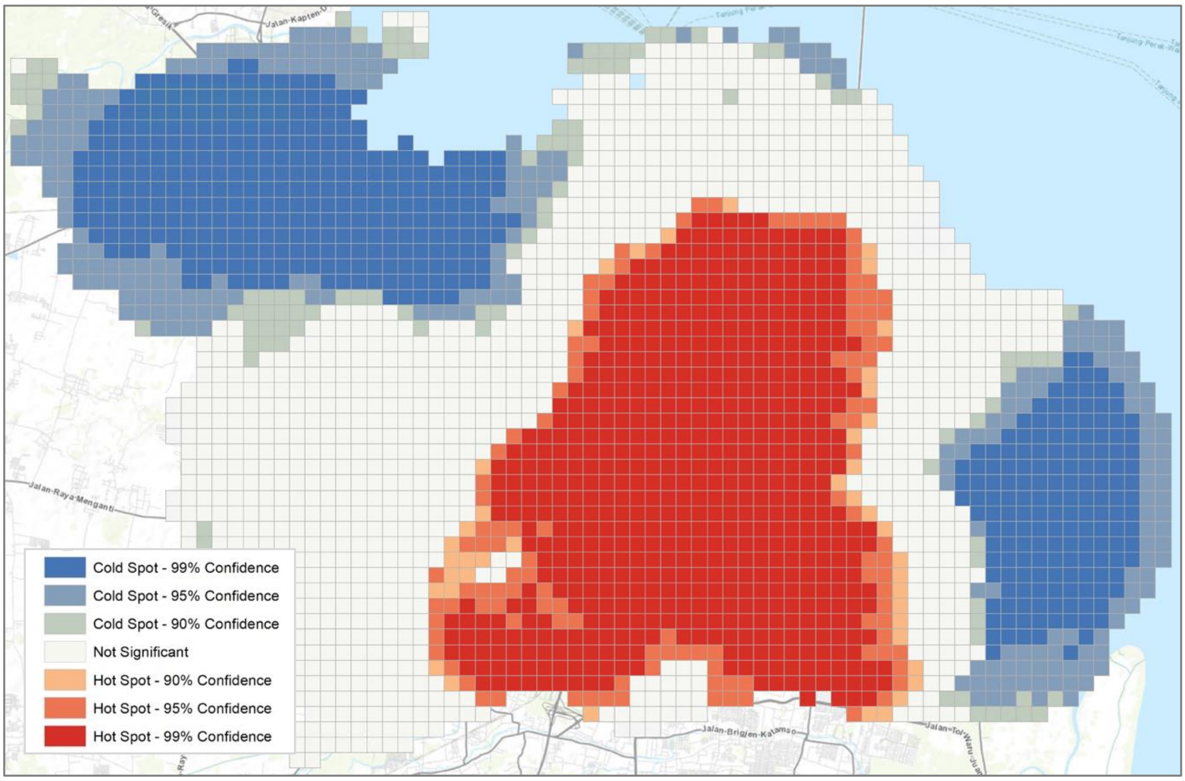

Note: Cold/hot spots are areas with significantly lower/higher density of outdoor tobacco advertisements. Hotspot analysis was conducted in ArcMap.

Appendix 3. Kernel density of outdoor tobacco advertisements in Surabaya 2018

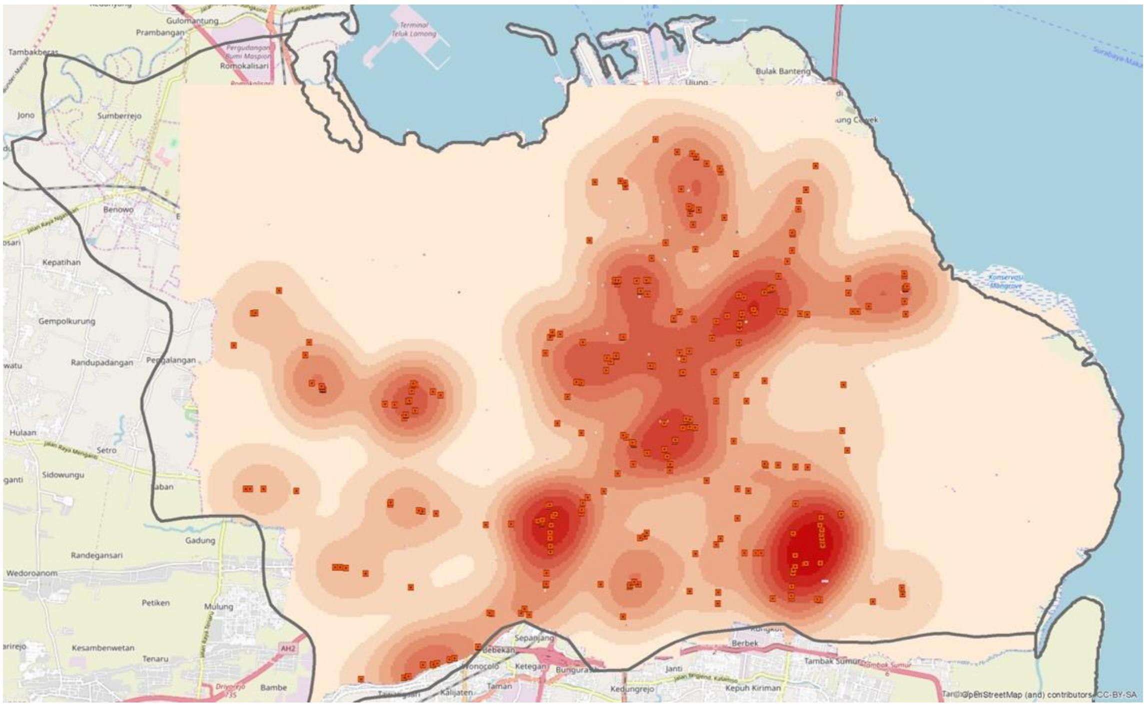

Note: Red squares show locations of outdoor tobacco advertisements. Kernel density used natural breaks and darker red color gradient shows denser advertisements.

Appendix 4. Overlay outdoor tobacco advert hotspots and subdistrict boundaries

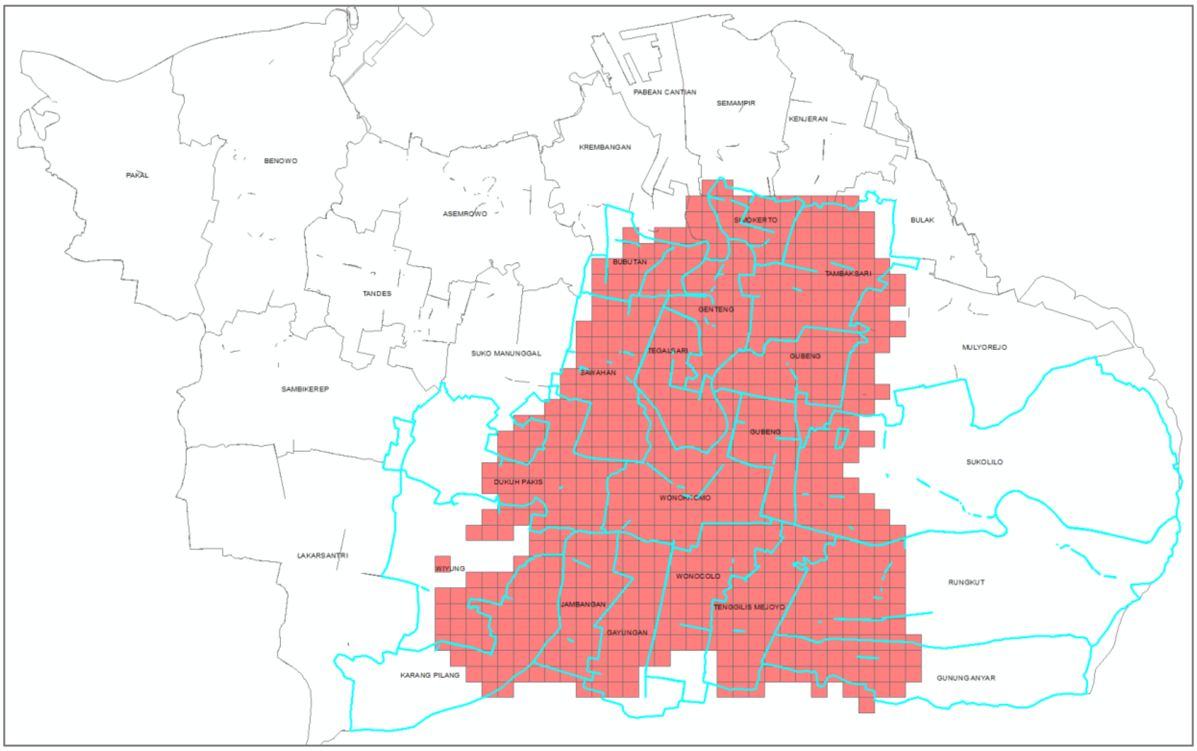

Note: The red square areas are hot spots of outdoor tobacco advertisements; the baseline map was removed to improve identifying the subdistrict boundaries.

Appendix 5. Characteristics of subdistricts and hotspot status

No	Subdistrict	City Region	Population	Area size km2	Density per km2	Poverty rate	Within hotspot
1	Bubutan	Central	106,721	3.9	27,648	38.4	Yes
2	Genteng	Central	62,028	4.1	15,316	7.9	Yes
3	Simokerto	Central	102,654	2.6	39,635	n/a	Yes
4	Tegalsari	Central	107,070	4.3	24,958	30.4	Yes
5	Gubeng	East	142,527	8.0	17,838	n/a	Yes
6	Tambaksari	East	233,502	9.0	25,974	15.6	Yes
7	Tenggilis Mejoyo	East	59,149	5.5	10,715	10.3	Yes
8	Dukuh Pakis	South	62,491	9.9	6,287	14.9	Yes
9	Jambangan	South	51,888	4.2	12,384	16.0	Yes
10	Sawahan	South	214,252	6.9	30,917	36.6	Yes
11	Wonocolo	South	83,735	6.8	12,369	n/a	Yes
12	Wonokromo	South	169,074	8.5	19,962	24.2	Yes
13	Gunung Anyar	East	57,806	9.7	5,953	n/a	No*
14	Mulyorejo	East	88,946	14.2	6,259	n/a	No*
15	Rugkut	East	115,501	21.1	5,479	n/a	No*
16	Sukolilo	East	113,551	23.7	4,795	n/a	No*
17	Karangpilang	South	75,433	9.2	8,173	n/a	No*
18	Wiyung	South	71,740	12.5	5,758	n/a	No*
19	Gayungan	South	47,286	6.1	7,790	1.1	No
20	Bulak	North	44,576	6.7	6,633	29.2	No
21	Kenjeran	North	167,031	7.8	21,497	9.6	No
22	Krembangan	North	12,375	8.3	1,484	21.0	No
23	Pabean Cantikan	North	85,069	6.8	12,510	n/a	No
24	Semampir	North	199,578	8.8	22,783	n/a	No
25	Asemrowo	West	48,188	15.4	3,121	41.5	No
26	Benowo	West	64,186	23.7	2,705	14.2	No
27	Lakarsantri	West	54,953	19.0	2,894	8.0	No
28	Pakal	West	58,804	22.1	2,664	11.0	No
29	Sambikerep	West	63,836	23.7	2,696	19.7	No
30	Sukomanunggal	West	104,932	9.2	11,369	n/a	No
31	Tandes	West	94,626	11.1	8,548	13.2	No
	TOTAL		2,963,508	332.6	8,909		

Note: Data were obtained from published reports by Surabaya Statistics Bureau (one city report and 31 subdistrict reports). Not all subdistrict reports have data on poverty rate (proportion of poor household), when not available, we summed the number of poorer categories (pra-sejahtera and sejahtera I) and divided by the number of household or population in each subdistrict. Hotspot status was based on the overlay of hotspots and subdistrict boundaries in Appendix 5; Yes = all or mostly within hotspot, No = outside hotspots, No = some subdistrict area was within hotspot.*

© 2019 Megatsari H.
